# Genome-Wide Identification and Expression Analysis of *BrBASS* Genes in *Brassica rapa* Reveals Their Potential Roles in Abiotic Stress Tolerance

**DOI:** 10.3390/cimb46070396

**Published:** 2024-06-28

**Authors:** Zhaojing Ji, Ruolan Wang, Meiqi Zhang, Luhan Chen, Yuexin Wang, Jiyun Hui, Shiya Hao, Bingcan Lv, Qiwei Jiang, Yunyun Cao

**Affiliations:** College of Horticulture Science and Engineering, Shandong Agricultural University, Tai’an 271018, China; 2022120394@sdau.edu.cn (Z.J.);

**Keywords:** *Brassica rapa*, *BASS*, gene family, bioinformatics analysis, abiotic stress

## Abstract

The bile acid sodium symporter (BASS) family plays an important role in transporting substances and coordinating plants’ salt tolerance. However, the function of *BASS* in *Brassica rapa* has not yet been elucidated. In this study, eight *BrBASS* genes distributed on five chromosomes were identified that belonged to four subfamilies. Expression profile analysis showed that *BrBASS7* was highly expressed in roots, whereas *BrBASS4* was highly expressed in flowers. The promoter element analysis also identified several typical homeopathic elements involved in abiotic stress tolerance and stress-related hormonal responses. Notably, under salt stress, the expression of *BrBASS2* was significantly upregulated; under osmotic stress, that of *BrBASS4* increased and then decreased; and under cold stress, that of *BrBASS7* generally declined. The protein–protein interaction analysis revealed that the *BrBASS2* homologous gene *AtBASS2* interacted with Nhd1 (N-mediated heading date-1) to alleviate salt stress in plants, while the *BrBASS4* homologous gene *AtBASS3* interacted with BLOS1 (biogenesis of lysosome-related organelles complex 1 subunit 1) via co-regulation with SNX1 (sorting nexin 1) to mitigate an unfavorable growing environment for roots. Further, Bra-miR396 (Bra-microRNA396) targeting *BrBASS4* and *BrBASS7* played a role in the plant response to osmotic and cold stress conditions, respectively. This research demonstrates that *BrBASS2, BrBASS4*, and *BrBASS7* harbor great potential for regulating abiotic stresses. The findings will help advance the study of the functions of the *BrBASS* gene family.

## 1. Introduction

Bile acid sodium symporter (BASS) proteins belong to the sodium-dependent transporter family and are classified as a solute carrier family 10 (SLC10). This family mainly facilitates the transport of substances such as bile acids and steroid hormones within cells [1]. The *BASS* gene family is found in many diverse organisms, such as fungi, bacteria, animals, and plants. In mammals, bile acid transporters in the liver and intestine play a crucial role in driving the enterohepatic circulation of bile acids [1], which promote the intestinal absorption of lipids and fat-soluble vitamins A, D, E, and K in humans [2]. Nevertheless, BASS family members from different species retain highly conserved Na-binding sites [3,4] to effectively participate in Na^+^ transport within organisms. Ranging in length from 348 to 477 amino acids, BASS proteins consist of 10 transmembrane fragments (TM1–10) and two conserved Na^+^ binding sites, Na1 and Na2 [5,6], and have a unique feature: two discontinuous helices that intersect at the center. Specific Na^+^ binding sites are typically located on these four transmembrane domains: TM3, TM4, TM5, and TM9 [4,7]. Specific binding of two sodium ions to the Na^+^ binding site is a prerequisite for structural changes in BASS and substrate binding. This arrangement enables the involvement of *BASS* gene family proteins in moving sodium ions during substrate transport to help balance ionic contents inside and outside the cell, thereby maintaining normal cellular function [6].

In plants, the *BASS* genes have been studied in several species, namely *Triticum aestivum* [8], *Gossypium hirsutum* [9], *Arabidopsis thaliana* [10], *Oryza sativa* [11], and species of the genera *Flaveria* and *Cleome* [12], showing them to figure prominently in plant responses to abiotic stressors (especially salt stress) and plant development. For example, overexpression of *TaBASS2* in *A. thaliana* and *O. sativa* can improve their respective salt tolerance; *TaBASS2* enhances the salt tolerance of *T. aestivum* by inhibiting the expression of ABI4, a node that links ABA (abscisic acid) signaling and plastid retrograde signaling pathways to the salinity response [8]. Moreover, *TaBASS2* overexpression in the *Atbass2* mutant also restored the sensitivity of *A. thaliana* to mevastatin [13]. In cotton (*G. hirsutum*), *GhBASS1* and *GhBASS3* positively regulate that plant’s salt content, while *GhBASS2*, *GhBASS4,* and *GhBASS5* negatively regulate its salt tolerance [6]; in this latter respect, *GhBASS5* operates by increasing Na^+^ transport and accumulation at the cellular and tissue levels [6,9]. Under salt stress conditions, the overexpression of *GhBASS5* in *A. thaliana* can significantly increase the Na^+^ content of that plant while strongly disrupting plastid function and enhancing salt sensitivity in transgenic *A. thaliana*. Under salt stress, *GhBASS5* overexpression lines significantly express salt-responsive transporter genes regulating K^+^/Na^+^ homeostasis [9].

In *A. thaliana*, *AtBASS1* is known to transport pantolytic acid in pantothenic acid synthesis, as demonstrated by expressing *AtBASS1* in yeast and constructing *Atbass1* mutants [14]. In addition, *AtBASS5* in chloroplasts acts as a carrier for 2-ketoacids and is involved in aliphatic glucosinolate (GLS) biosynthesis [10,15], while the AtBASS6 protein transports glycolic acid in the photorespiratory metabolism of *A. thaliana* [16]. In rice (*O. sativa*), OsSBF1 shows pronounced homology with bile acid transporters in both mammals and bacteria and is possibly involved in the transport of sulfonated brassin-steroids. In studies of *Flaviria* and *Cleome*, *BASS2* from *A. thaliana* was highly expressed in both C_4_ plants and localized to their chloroplast envelope, providing molecular-level evidence for sodium-coupled metabolite transporters in the plastid envelope [12].

*Brassica* plants, including *B. Juncea*, *B. Oleracea* and *B. napus*, and *B. rapa*, are globally important crops known for their diversity. Chinese cabbage, a *B. rapa* subspecies, is particularly valued for its economic and nutritional value to humans. However, it is subject to various biotic (e.g., fungi, bacteria, viruses, insects) and abiotic stresses (e.g., low temperature, salt, drought) in its natural environment. This leads to severe growth stagnation, yield loss, and quality reduction of Chinese cabbage crops [17,18,19]. Current research on the *BASS* gene family has mainly focused on substrate binding and transport mechanisms. However, the function and regulation of *BASS* genes in Chinese cabbage have not been fully investigated. Accordingly, an analysis of sequence characteristics, gene expression patterns, regulatory mechanisms, and epigenetics of *BrBASS* genes was carried out in this study. These results are important for future research on abiotic stressors of the *BrBASS* gene and for the development of stress-tolerant cabbage varieties.

## 2. Materials and Methods

### 2.1. Identification and Physicochemical Characterization of BrBASSs

*B. rapa* and *A. thaliana* whole genome sequences, gff3 genome annotation data, and *BASS* amino acid sequences were downloaded from EnsemblPlant (http://plants.ensembl.org/, accessed on 18 February 2024). The *BrBASS* genes were screened from the *B. rapa* genome, using the BLASTP program with amino acid sequences of *AtBASS* serving as input, to predict the *BrBASS* genes. All *B. rapa* BASS proteins were predicted by using Expasy technologies (http://www.expasy.org/, accessed on 18 February 2024) and their subcellular distribution was analyzed using the WOLF PSORT website (https://www.genscript.com/wolf-psort.html, accessed on 15 March 2024) [20,21].

The amino acid sequences of *BrBASSs* and *AtBASSs* were aligned using the MUSCLE algorithm in MEGA-X software (v10.0.1) [22]. A maximum likelihood phylogenetic tree was then constructed using MEGA X (with 1000 bootstrap replicates). The resulting phylogenetic tree was modified for a clearer presentation, using iTOL (https://itol.embl.de/, accessed on 17 March 2024) [23]. Synteny was analyzed with the ‘Advanced Circles and Table Row Extract’ or ‘Filter’ programs of TBtools software (v1.120) [24].

### 2.2. Gene Structure and Conserved Domain Analysis

Each *BrBASS* was visualized using the Visualize Gene Structure program of TBtools (v1.120), and their conserved domains were analyzed using NCBI’s CD-Search tools (v3.18) (http://www.ncbi.nlm.nih.gov/cdd, accessed on 19 February 2024). The conserved motifs of the identified *BrBASSs* were analyzed with the online tool MEME (v5.5.1) (http://meme-suite.org/tools/meme, accessed on 19 February 2024), for which the predicted number of motifs was set to 10.

### 2.3. Analysis of Promoter Cis-Elements of BrBASSs

The upstream 2000-bp sequence of each *BrBASS* was extracted with TBtools (v1.120), and the eight genes were then analyzed via the PlantCARE online website (https://bioinformatics.psb.ugent.be/webtools/plantcare/html/, accessed on 19 February 2024) [25]. The findings from this website’s analysis were summarized in MS Excel 2019 to present the results.

### 2.4. Tissue-Specific Expression of BrBASS Genes and Analysis of MicroRNAs Targeting the Genes of BrBASSs

Transcriptome sequences from various *B. rapa* tissues were obtained from the NCBI GEO (record number GSE43245) [26]. These sequences were normalized using Transcripts Per Million (TPM). The HeatMap plug-in of TBtools (v1.120) was utilized to create expression maps, and we collected the CDS sequences of *BrBASS* genes. MiRNAs targeting *BrBASS* genes were identified using the psRNATarget online database (accessed on 19 February 2024; https://www.zhaolab.org/psRNATarget/). These data were then visualized in MS Excel 2019 tables [27].

### 2.5. Plant Material and Stress Treatments

Stress treatments were applied to *B. rapa* plants that have stable self-incompatibility. These plants were grown in an incubator using Murashige and Skoog (MS) Modified Medium (Qingdao Hope Bio-technology, Qingdao, China), which included vitamins, sucrose, and agar. When their true leaves had expanded, the seedlings were transplanted into substrate-filled seedling pots and cultured in a plant incubator (16-h light/8-h dark photoperiod at 25 °C; light intensity of 2000 l×; relative humidity of 67%). Seedlings having six leaves and a similar growth status were then chosen to receive the stress treatments. In a hydroponic system, these seedlings were inserted into a mixture of 150 mM NaCl to simulate salt stress or 15% PEG 6000 (0.15 g/mL) to simulate drought conditions. Plants were exposed to 4 °C for the cold stress treatment. Unstressed seedlings from the same batch and of similar size served as the control (CK). Each stress treatment was applied for durations of 4, 6, and 12 h. Each treatment included three biological replicates; all samples were cryopreserved at –80 °C for subsequent RNA extractions.

### 2.6. Total RNA Extraction and RT-qPCR

The FastPure^®^ Cell/Tissue Total RNA Isolation Kit V2 (Vazyme Biotech Co., Ltd., Nanjing, China) was used to isolate total RNA from each replicate plant. TransScript Uni all-in-one first-strand cDNA synthesis SuperMix TransGen (AU341-02, Beijing, China) was used for the qPCR; for the RT-qPCR analysis, samples were subjected to reverse transcription. The RT-qPCR primer sequences were designed using the qPrimerDB-qPCR primer database (https://biodb.swu.edu.cn/qprimerdb/, accessed on 24 June 2024). Each RT-qPCR run was conducted on a CFXopus 96 qPCR machine (California, USA) by using the ChamQ SYBR qPCR premix (Nanjing, China) with *BrGAPDH* serving as the reference gene and applying the 2^−ΔΔCt^ method. Table S5 lists the specific primer sequences used in the experiments that were conducted in technical triplicates.

### 2.7. Statistical Analysis 

The determination of significant differences between means was made using two-tailed *t*-tests, implemented in IBM SPSS 25 (significance level, α = 0.05).

### 2.8. BASS Protein Secondary Structure and Tertiary Structure Analysis 

For the protein secondary structure analysis, we utilized the predict protein tool (https://predictprotein.org/, accessed on 19 February 2024). The results were analyzed and plotted using MS Excel 2019. To predict the tertiary structure of each BrBASS protein, we used SWISS-MODEL (v1.0) (https://swissmodel.expasy.org/, accessed on 19 February 2024).

### 2.9. Predicted Protein–Protein Interactions of BrBASSs and Phosphorylation Site Analysis

For the protein interaction network analysis, we employed the protein interaction network prediction website (https://cn.string-db.org/, accessed on 17 March 2024) to obtain *B. rapa* protein network interaction maps (minimum interaction score required = 0.150; other parameters used at their default settings). To examine the BrBASS protein interaction network, the STRING online server (https://string-db.org/cgi, accessed on 17 March 2024) was used under its default parameters [28]. The interaction network was constructed in Cytoscape v3.9.1 [29]. *BrBASS* gene phosphorylation sites were analyzed using the online site Netphos 2.0, with their visual representations prepared in MS Excel 2019.

## 3. Results

### 3.1. Identification and Physicochemical Characterization of BrBASS Family Genes

To analyze the basic features of the *BrBASS* gene family, we screened the cabbage genome for six amino acid sequences of *AtBASSs* through HMMER and Blast. Eight members of the *BrBASS* gene family were finally obtained and renamed *BrBASS1*–*BrBASS8* (Table 1). Relevant bioinformatics analyses showed that the amino acid lengths of *BrBASSs* ranged from 212 aa (*BrBASS8*) to 440 aa (*BrBASS4*), with a molecular weight size of 22,989.89 Da (*BrBASS8*) to 46,419.66 Da (*BrBASS4*). Their isoelectric points spanned 8.95 (*BrBASS5*) to 9.87 (*BrBASS3*), indicating that all eight members of the *BrBASS* family were basic proteins. Their subcellular localization showed them distributed in various cytoplasmic compartments within the cell, including the chloroplast, plastid, endoplasmic, reticulum, mitochondrion, cytosol, vascular, cytosol, etc. Still, all eight *BrBASS* genes’ proteins were located in the chloroplast, suggesting they may regulate vital cellular activity in a specific manner.

### 3.2. Phylogenetic Relationships and Synteny Analysis

**Figure 1:**
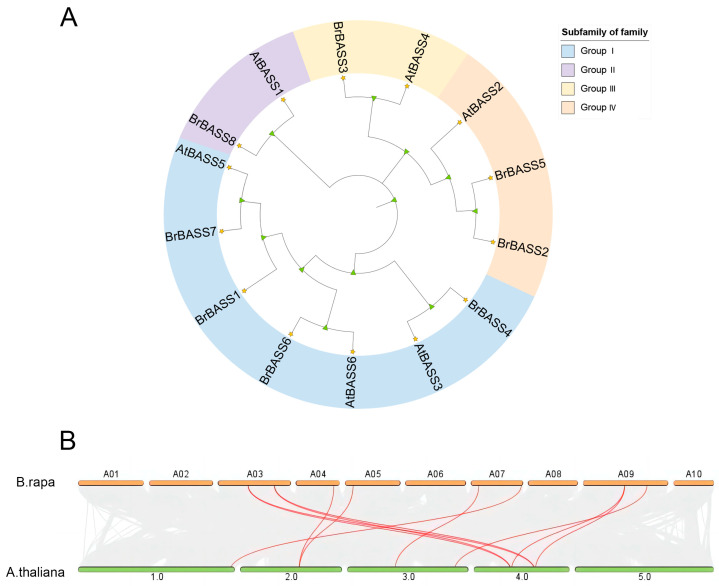
(**A**) Evolutionary tree of the *BASS* family in *B. rapa* and *A. thaliana*. The triangles represent leaf nodes, and the five-pointed stars represent branch nodes. (**B**) Collinearity analysis between *B. rapa* and *A. thaliana*. Orange boxes represent the 10 chromosomes of *B. rapa*, and green boxes represent the five chromosomes of *A. thaliana*. Red lines indicate homologous gene pairs.

The phylogenetic analysis of *BrBASSs*, along with the *BASSs* in *A. thaliana*, was carried out using the maximum likelihood method (Figure 1A). This analysis revealed that the 14 *BASSs* clustered into four major groups (I, II, III, IV) based on their evolutionary relationships. According to their evolutionary relationships, Group I was the largest, comprising seven members: four *B. rapa* genes and three *A. thaliana* genes. Group II consisted of just two members: one from *B. rapa* and one from *A. thaliana*. Group III also had two *BASS* members: one each from *B. rapa* and *A. thaliana*. Group IV contained three members: two *B. rapa* genes and one *A. thaliana* gene.

The collinear analysis between *B. rapa* and *A. thaliana* (Figure 1B, Table S1) detected strong collinearity between six *BrBASSs* and five genes in *A. thaliana*.

### 3.3. Gene Structure and Conserved Domain Analysis

**Figure 2:**
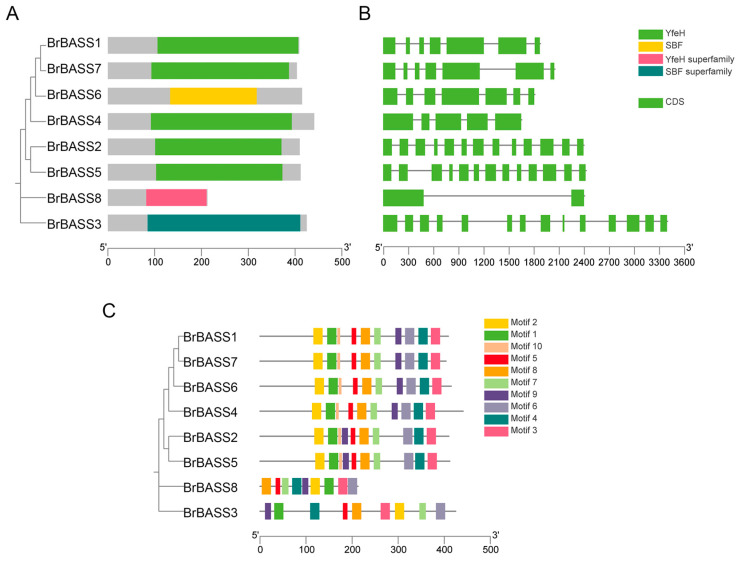
Gene structure and conserved domain analysis of *BrBASSs*. (**A**) Analysis of conserved domains of *BrBASS* genes. (**B**) Analysis of their gene structure. The introns and exons are shown as black lines and green boxes, respectively. (**C**) Analysis of the conserved motifs of *BrBASS* genes.

We used NCBI-CDD to predict the conserved domains of *BrBASSs* (Figure 2A). This showed that the *BrBASSs* encoded four protein domains, namely YfeH, SBF, the YfeH superfamily, and the SBF superfamily. Notably, *BrBASS3* and *BrBASS8*, respectively, contained the SBF and YfeH superfamilies, suggesting that members of these superfamilies may have special functions. Only *BrBASS3* and *BrBASS6* contained the SBF domain, and the other six family members all harbored the YfeH domain.

Genetic structure analysis revealed 2 to 14 exons in the *BrBASS* genes (Figure 2B). The protein-conserved motifs of *BrBASS* genes were analyzed via the MEME website (Figure 2C), with a total of 10 motifs identified. These results showed that most of the motifs had a similar composition, and all contained conserved Motif 1, likely the core motif of the *BrBASS* family. Except for *BrBASS3* and *BrBASS8*, which had nine motifs, the other genes had 10 motifs. In the same clade, conserved motifs are similar in number, type, and arrangement; hence, functional differences among the *BrBASS* genes could be due to a different distribution of conserved motifs, so we divided them into four different groups. These findings were consistent with our gene structure analysis and confirmed the subfamily division of the *BrBASS* genes.

### 3.4. Analysis of Promoter Cis-Elements of BrBASSs

**Figure 3:**
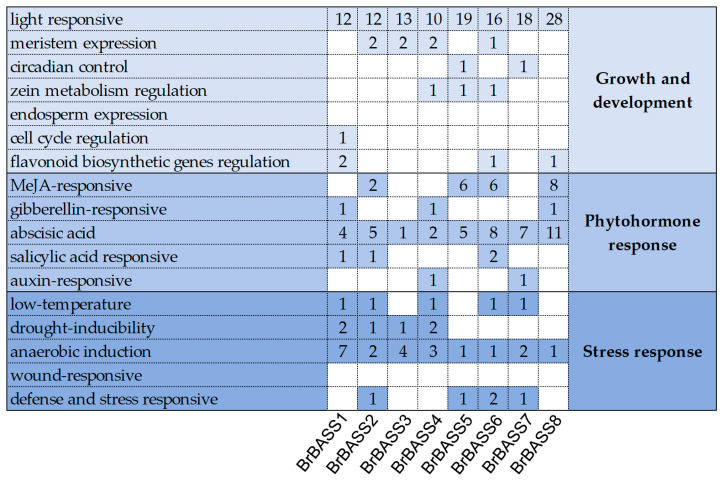
Analysis of *cis*-elements in the promoter region of the eight *BASS* genes in *B. rapa.* Different shades of blue indicate the presence of *cis*-elements involved in different biological processes; the number in each cell indicates the number of *cis*-acting elements for that gene. White cells indicate the absence of the *cis*-acting element.

To better understand their gene expression patterns, we used PlantCARE to analyze the 2000-bp sequence upstream of each *BrBASS* gene (Figure 3). Concerning those related to growth and development, all eight *BrBASS* genes contained light-responsive elements but were devoid of endosperm expression and wound-response elements. Among phytohormone-responsive elements, all the genes contained abscisic acid elements; 50% of them contained methyl jasmonate (MeJA)-responsive elements; and 37.5% of them contained gibberellin-responsive and salicylic acid-responsive elements. Among the stress response-related elements, all the genes contained anaerobic induction elements; 62.5% of them contained low-temperature elements; and 50% of them contained drought-inducibility elements or defense and stress-responsive elements.

### 3.5. Analysis of Tissue-Specific Expression of BrBASSs

**Figure 4:**
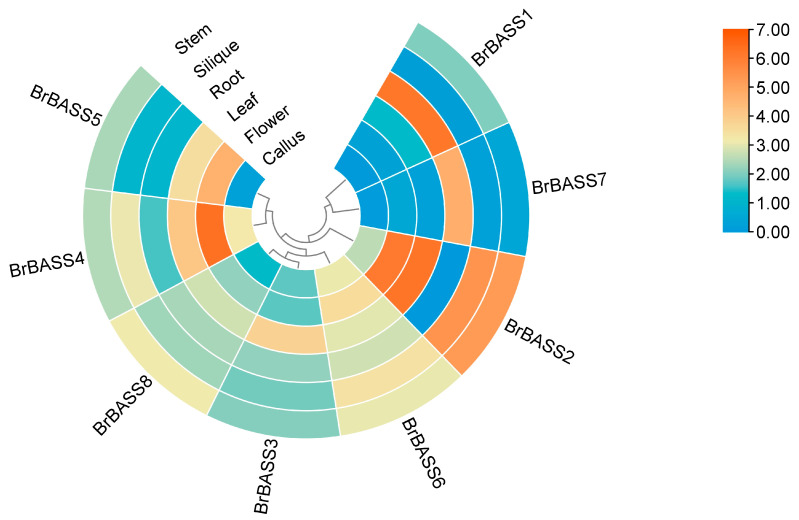
Tissue-specific expression levels of the eight *BrBASS* genes. TPM was used to normalize the transcriptome sequences in different *B. rapa* tissues (from the NCBI GEO, accession number: GSE43245). The heatmap shows the level of *BrBASS* genes’ expression across six plant tissues. The red-to-blue color gradient indicates higher to lower expression levels.

To understand the tissue-specific expression patterns of *BrBASS* genes, we examined the transcriptome data obtained from various tissues (Figure 4, Table S2). This revealed that *BrBASS2* and *BrBASS4* were expressed at particularly high levels in flowers, which might be related to their developmental process and potential role in sexual reproduction. Crucially, *BrBASS1* and *BrBASS7* were distinguished by high expression levels in the roots, which indicated their potential functionality in responding to abiotic stressors (such as salt and osmotic stresses). In addition, *BrBASS2* was significantly overexpressed in leaves, which pointed to its possible regulatory role under low- and high-temperature stress conditions.

### 3.6. Analysis of Expression Patterns in Response to Abiotic Stress

**Figure 5:**
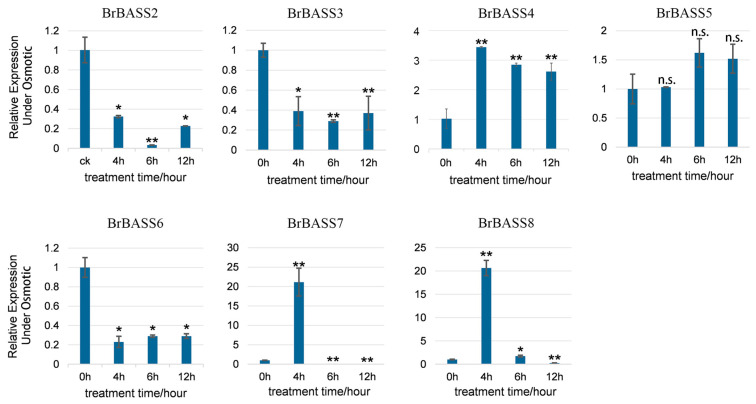
Expression profiles of seven *BrBASS* genes under osmotic stress from the RT-qPCR analysis. The above experiments were performed using 0 h as the control, with the treatment time set to 4, 6, and 12 h. Each group had three biological replicates, and the error bars represent the standard error of the mean. Asterisks indicate statistically significant differences (two-tailed *t*-test; * *p* < 0.05; ** *p* < 0.01; n.s., no significance).

Chinese cabbage is very susceptible to osmotic stress, cold stress, and salt stress during its growth. So, we selected seven genes for RT-qPCR detection under conditions of osmotic stress, cold stress, and salt stress to distinguish which *BrBASS* genes were associated with each form of abiotic stress. Both *BrBASS4* and *BrBASS8* always exhibited downregulated expression under either osmotic, low temperature, or salt stress. However, as Figure 5 shows, under osmotic stress, the relative expression levels of *BrBASS2*, *BrBASS3,* and *BrBASS6* decreased significantly, while those of *BrBASS4*, *BrBASS7*, and *BrBASS8* increased at first but then decreased, peaking at 4 h and 6 h, respectively.

In addition to these genes, we also analyzed the expression of *BrBASS1* under the same stress conditions. The results showed that the expression level of *BrBASS1* remained consistently low and did not exhibit significant changes across all the time points studied. This suggests that *BrBASS1* may not play a significant regulatory role under osmotic, cold, or salt stress conditions. Furthermore, its homologous gene in *A. thaliana*, *BASS5*, has not been extensively studied under stress conditions. Therefore, *BrBASS1* was not included in the detailed analysis and discussion of stress-responsive genes.

**Figure 6:**
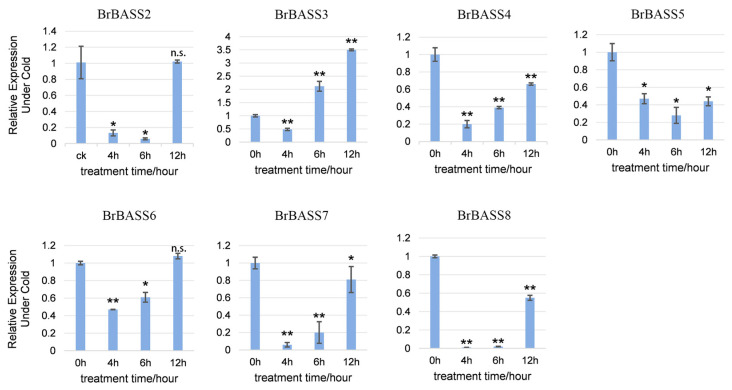
Expression profiles of seven *BrBASS* genes under cold stress from the RT-qPCR analysis. The above experiments were performed using 0 h as the control, with the treatment time set to 4, 6, and 12 h. Each group had three biological replicates, and the error bars represent the standard error of the mean. Asterisks indicate statistically significant differences (two-tailed *t*-test; * *p* < 0.05; ** *p* < 0.01; n.s., no significance).

Under cold stress (Figure 6), except for *BrBASS3*, whose relative expression significantly exceeded that of the control (CK) by three times, all the genes (*BrBASS2*, *BrBASS4*–*BrBASS8*) showed declines in their expression, with *BrBASS2*, *BrBASS7*, and *BrBASS8* decreasing the most at 4 h and 6 h. The relative expression of *BrBASS5* was significantly downregulated as well. 

**Figure 7:**
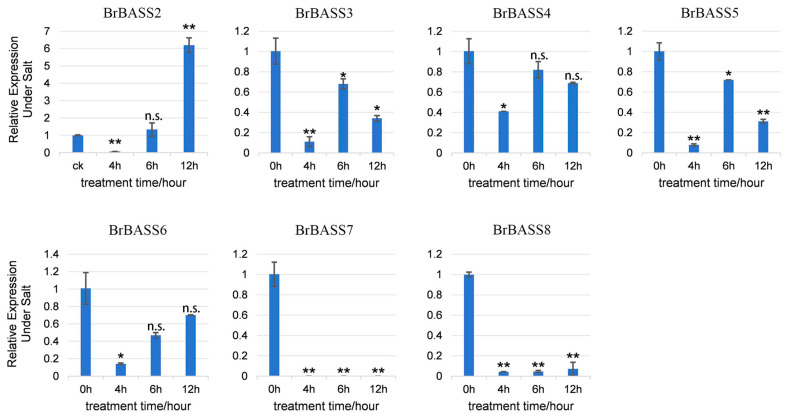
Expression profiles of seven *BrBASS* genes under salt stress from the RT-qPCR analysis. The above experiments were performed using 0 h as the control, with the treatment time set to 4, 6, and 12 h. Each group had three biological replicates and the error bars represent the standard error of the mean. Asterisks indicate statistically significant differences (two-tailed *t*-test; * *p* < 0.05; ** *p* < 0.01; n.s., no significance).

Under salt stress (Figure 7), except for the drastically increased relative expression levels of *BrBASS2* and *BrBASS7*, which were basically reduced to 0, the other five genes’ relative expression levels showed differing degrees of downregulation, this being most pronounced at 4 h. With a longer treatment time, their relative expression increased but was still lower than that of CK. It is worth noting that *BrBASS2* was significantly downregulated at 4 h, and its relative expression level showed an opposite trend with prolonged treatment time, peaking at 12 h, which was six times that of CK.

### 3.7. Protein Secondary Structure and Tertiary Structure Prediction of BrBASSs

**Figure 8:**
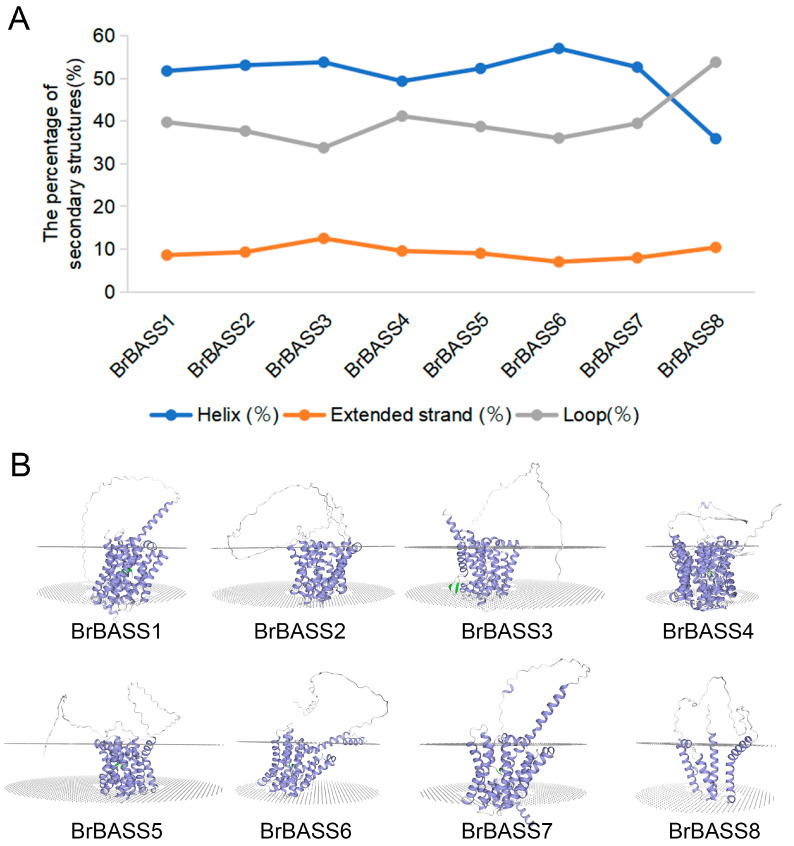
Protein secondary and tertiary structure prediction of BrBASSs. (**A**) Predicted secondary structure of BrBASS proteins. Different colors indicate different secondary structures. (**B**) Predicted tertiary structures of BrBASS proteins. The light blue band represents the alpha helix in the BASS protein structure; the silver-white band indicates irregular coiling; and the green portion indicates the non-helical part of the discontinuous helix. The portion between the dotted planes is the transmembrane region of the protein under prediction.

The structure of proteins is linked to their biological functions, so studying the structure of BASS proteins can provide valuable insights into their functions. Here, we analyzed the predicted protein secondary structures of BrBASSs and found that all members had helixes, extended strands, and loops. The protein secondary structures of BrBASSs are mainly helixes, whereas extended strands are least abundant (Figure 8A). According to the SWISS-MODEL analysis, members of the same subgroup displayed homologous protein tertiary structures (Figure 8B), indicating they have maintained homologous structures during evolution. BrBASS proteins consist of multiple transmembrane fragments (TM helixes) and two conserved sodium-binding sites, Na1 and Na2 (Figure 8B and Figure S1), located on the transmembrane structural domains [4,5,6,7]. The two discontinuous helices intersect in the center: a unique structural feature that enables BrBASS proteins to bind sodium ions during substrate transport and to enact structural changes by specifically binding to the sodium-binding site. This binding of sodium ions is a prerequisite for the binding and transport of BASS proteins to substrates, and, through the movement of sodium ions, this helps to balance the ionic content inside vs. outside the cell and to maintain normal cellular functions [6]. This structure allows BrBASS proteins to play a pivotal role in transmembrane transport dynamics by regulating the movement of sodium ions to facilitate the efficient transport of substrates, thus figuring prominently in the growth and responses of plants to environmental stress. Altogether, this provides basic information that could be helpful for subsequent studies of the functions of BrBASS proteins.

### 3.8. Predicted Protein–Protein Interactions

**Figure 9:**
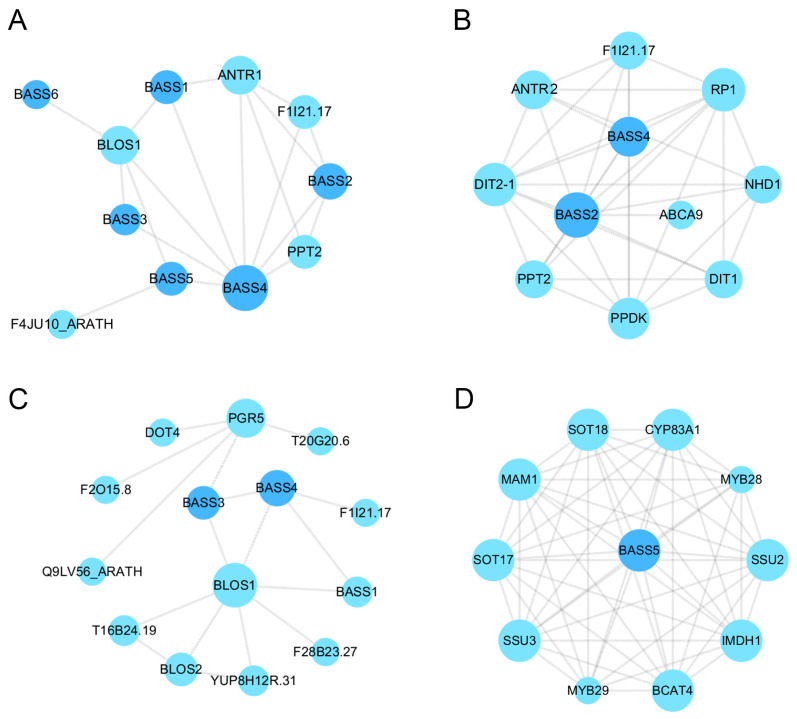
Predictive PPI interaction networks for BASSs in *A. thaliana*. (**A**) Predictive analysis of BASS interaction networks in *A. thaliana*. (**B**) Predictive analysis of BASS2 in *A. thaliana*. (**C**) Predictive analysis of the interaction network of BASS3 in *A. thaliana*. (**D**) BASS5 interaction network prediction in *A. thaliana*. The minimum engagement score requirement is 0.150, and all other parameters were set to default. Nodes represent proteins, and edges represent protein-protein interactions.

Proteins are essential for the execution of myriad cellular and tissue functions and are implicated in diverse life activities [30]. PPI networks consist of protein interactions involved in various life processes, including biological signaling, gene expression regulation, regulation of the cell cycle, and intricate coordination of energy and material metabolism [31,32,33,34]. It is possible to gain a deeper understanding of the complex biological functions of proteins and biomolecular interactions by identifying connections between unknown functional proteins and PPI interaction networks [35,36]. The integrated resources and algorithms in the STRING database were utilized in our construction of predicted PPI network maps for detecting strong interactions between BASS members in *A. thaliana*, namely between BLOS1 and ANTR1 (sodium-dependent phosphate transport protein 1) (Figure 9A, Table S3). *BrBASS1* and *BrBASS7* were orthologous to *AtBASS5,* while *BrBASS2* was orthologous to *AtBASS2*.

BLOS1 possibly regulates PIN1 (PIN-formed 1) and PIN2 (PIN-formed 2) homeostasis through its interaction with SNX1 in auxin-mediated root growth and development of *A. thaliana* [30,37,38].

The analysis of the protein-protein interaction (PPI) network uncovered a notable resemblance in the anticipated PPI networks of BASS2, 3, and 5 in *A. thaliana* and the proteins encoded by the *BrBASS2* and *BrBASS3* homologs (*AtBASS2* and *AtBASS3*, respectively), which were found to interact with the abovementioned Nhd1 and BOLS1 in *A. thaliana*, respectively (Figure 9B,C). Nhd1 functions as a chloroplast sodium exporter, and its transport activity plays an important role in protecting vital chloroplast reactions such as photosynthesis from toxicity due to high sodium levels [39]. Furthermore, MYB (myeloblastosis) transcription factors are involved, including MYB28 and MYB29, which interact with the proteins encoded by homologs of *BrBASS5* (Figure 9D). *Brassica* plants respond to abiotic stresses via proteins encoded by *BrBASSs*, which interact with proteins encoded by different gene families.

### 3.9. Phosphorylation Site Analysis

**Figure 10:**
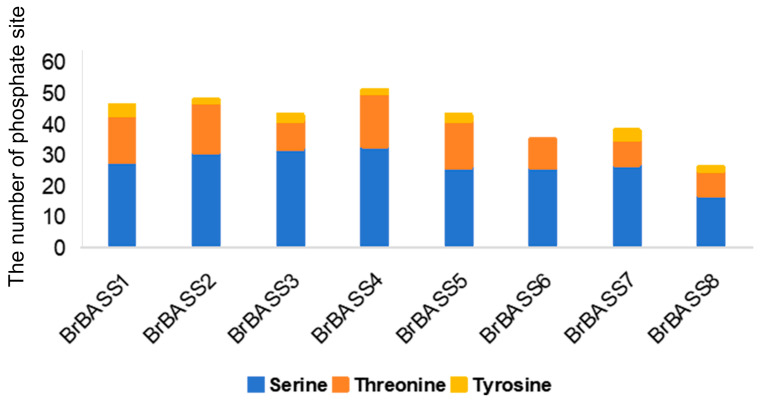
Distribution of predicted phosphorylation sites in the amino acid sequences of BrBASSs.

Protein phosphorylation modifications play a key role in signal transduction in the development and environmental adaptation of plants [40]. Protein phosphorylation mainly occurs on serine, threonine, and tyrosine residues [41]. To identify these processes—better known as protein phosphorylation—in BrBASSs, we conducted a phosphorylation site analysis (Figure 10). All proteins encoded by the eight members of the *BrBASS* gene family contained phosphorylation sites. We uncovered 330 phosphorylation sites, of which 66.7% were serine residues, 29.3% were threonine residues, with just 3.93% accounted for by tyrosine residues. Further examination revealed that all of the *BASS* genes in *B. rapa*, except *BrBASS6*, contained phospho-serine, phospho-tryptophan, and phospho-tyrosine sites. It should be noted that *BrBASS4* harbored the highest number of phosphate sites.

### 3.10. Prediction of microRNAs Targeting BrBASSs

MicroRNAs (miRNAs) regulate their target genes post-transcriptionally in a negative manner [42,43]. Since they can have important functions in regulating gene expression, we investigated the miRNAs associated with *BrBASSs* genes (Table 2 and Table S4). These results revealed a total of 11 miRNA types regulating the eight *BrBASS* genes. *BrBASS4*, *BrBASS5*, and *BrBASS8* were targeted by differing miRNAs, and *BrBASS6* was the target of two different miRNAs. Notably, *BrBASS3* and *BrBASS7* were both targeted by three different types of miRNAs.

## 4. Discussion

Bile acid sodium symporter (BASS) family proteins are a class of Na^+^/solute isotropic transporters. Several studies have revealed that the *BASS* gene family is instrumental in sodium transport activities in mammals and for mediating how plants adapt to abiotic stresses, especially in coordinating their salt tolerance traits [5,6,8], but its function has not been systematically studied in *B. rapa*. In the present study, eight *BrBASS* genes were identified and obtained, with the aim of elucidating their role in abiotic stress response by analyzing not only the structure and expression pattern of this family but also their physicochemical properties, gene structure, and *cis*-acting elements, etc. This study deepens our understanding of the functioning of *BrBASSs*.

Collinearity analysis can help to infer local events based on incongruity with the surrounding environment, often resulting in gene conversion [44]. When applied here to *BrBASSs* and *AtBASSs*, this analysis uncovered as many as seven groups of collinear genes between both, involving six *BrBASSs* (75% of the entire *BrBASS* gene family) and five *AtBASSs* (83.3%). This indicates that the evolutionary relationship between the *BrBASS* gene family and the *AtBASS* gene family is quite close. Accordingly, the functional study of *AtBASS* genes can provide a key reference for the functional research of *BrBASS* genes.

In *B. rapa*, the subcellular localization of its *BrBASS* gene family shows that all eight genes are distributed on chloroplasts. As a plastid, chloroplasts are not only the primary site for the excessive production of reactive oxygen species (ROS) in response to abiotic stresses [45,46], but also an important site for the production of antioxidant enzymes (e.g., superoxide dismutase (SOD), catalase (CAT), and peroxidase (POD)) to mitigate ROS toxicity [45,47]. In plants, ROS metabolism is a crucial component of their responsive adaptation to osmotic, cold, and salt stresses [48,49,50]. Many studies have suggested that chloroplasts may be important in plant responses to abiotic stress conditions [51,52,53,54,55,56]. Under cold stress, chloroplasts can sense low-temperature signals via photoreceptors and membranes and promote photosynthesis by regulating enzyme activity and hormonal balance, thereby enhancing plant tolerance to cold stress [57]. SAL1-PAP chloroplasts in plants retrograde in response to nuclear stress, while the closure of stomata in leaves is responsive to osmotic stress [58]. Hence, we speculate the *BrBASS* gene family plays a key role in the abiotic stress dynamics of *B. rapa* plants.

*BrBASS4* may contribute to coping with osmotic stress. The RT-qPCR results showed that this gene’s expression was higher at 4 h and peaked at 6 h under the osmotic treatment. But at 12 h, the expression level of *BrBASS4* was significantly lower than that of CK. Analysis of its promoter *cis*-elements showed that *BrBASS4* contained drought-responsive elements as well as gibberellin-responsive, abscisic acid, and auxin-responsive elements associated with hormone regulation in plants. Gibberellin (GA) is a key phytohormone that enables plants to cope with osmotic stress via signal transduction pathways and active oxygen clearance. The biosynthesis and degradation of GA in the early stage of osmotic stress alters the stomatal opening of plants, which in turn affects the rate of transpiration; hence, it has been postulated that GA regulates plants’ osmotic tolerance by reducing leaf transpiration [59]. Under osmotic conditions, plants synthesize ABA to regulate the plasma membrane ion channels of guard cells, leading to the long-term outflow of negative ions and K^+^, which in turn causes the guard cells to shrink and the stomata to close, enabling plants to better cope with osmotic stress [60]. The ABA response element (ABRE) enhances plant tolerance by regulating gene expression under drought and osmotic stress conditions [61]. Furthermore, the key node of the DELLA protein may jointly regulate osmotic stress response in plants between GA signaling and ABA signaling [62]. It has been shown that the auxin-responsive factor (ARF) can bind directly to the promoter of auxin-responsive genes, thereby augmenting tomato’s response to osmotic stress [63].

Since *BrBASS4* contains the most serine and threonine residues, we speculate that the biological function of this gene may be determined by the phosphorylation of serine residues. This would play a key role in cell signal transduction [64], thus providing evidence for the osmotic stress response of the *BrBASS4* gene. According to the PPI results, *AtBASS3*, the homologous gene of *BrBASS4*, interacted with both BLOS1 and PGR5 (proton gradient regulation 5). In Arabidopsis, there is evidence showing that BLOS1 figures prominently in the response to osmotic stress. BLOS1 is capable of interacting directly with SNX1 and regulates the homeostasis of PIN1 and PIN2 by mediating vacuolar degradation transport [30]. This regulation affects the distribution of auxin in roots, which in turn modulates the process of cell division and expansion [25], allowing them to grow more actively under favorable soil conditions and to better tolerate arid conditions [65]. In addition, prediction of the target gene showed that Bra-miR396 targeted *BrBASS4*. Research using *A. thaliana* has found that miR396 is responsive to osmotic and salinity [66], and that overexpression of miR396 in tobacco confers osmotic tolerance [67]. Thus, we speculate that *BrBASS4* plays an important role in coping with osmotic stress.

*BrBASS7* harbors great potential for regulating cold stress. The RT-qPCR showed that this gene’s relative expression fell considerably under low temperature stress. Analysis of the promoter elements showed that *BrBASS7* contained a low-temperature response element, an abscisic acid, and an auxin-responsive hormone response element. When plants are exposed to cold conditions, stomatal opening in their leaves is inhibited, resulting in the accumulation of ABA during stomatal closure that in turn enables plants to cope with cold stress [50]. When *A. thaliana* is subjected to cold stress, the auxin transport rate is affected, allowing this plant to endure cold stress [68].

The target gene prediction analysis showed that Bra-miR396, Bra-miR9565, and Bra-miR5725 all targeted *BrBASS7*. A recent study of Jerusalem artichoke found that htu-miR396 was upregulated under cryogenic stress [69]. Analysis of the PPI network has revealed that *AtBASS5*, a homologous gene of *BrBASS7*, interacts with MYB28 and MYB29 proteins; MYB28 is a positive regulator of aliphatic methionine-derived glucosinolates (GLS), and MYB29 is involved in hormone-mediated pathways and signal transduction, playing a role in GLS biosynthesis and promoting it in conjunction with proteins such as MYB28/HAG1 [70,71]. Other research has confirmed that the synthesis of GLS in *Brassica* plants was also affected by ambient temperature, in that low temperatures promoted the production of GLS in response to external factors [72]. However, since *BASS5* was a candidate transporter gene involved in GLS synthesis in *A*. *thaliana*, it was posited that *BASS5* may promote GLS biosynthesis via its regulation at low temperatures [10]. In other words, *BASS5* could also be related to cold stress.

Salt stress could be alleviated by *BrBASS2.* The economic productivity of vegetable crops is greatly limited by salinity stress, being one of the most important environmental constraints [73]. The RT-qPCR results show that the relative expression of *BrBASS2* is upregulated under conditions of salt stress. The promoter *cis*-acting element of *BrBASS2* contains abscisic acid-responsive elements, MeJA-responsive elements, and salicylic acid-responsive elements. In cotton, ABA was continuously upregulated under salt stress, which implied its potential pertinence for mitigating that type of stress, and ABA’s accumulation underpinned the salt tolerance mechanism [74]. MeJA plays an important role in resisting salt stress, osmotic stress, cold stress, heavy metal stress, and the toxicity of other elements. After applying MeJA to stressed plants, they grow better, accumulate active compounds, and begin to undergo changes in their physiological and biochemical properties as well as their endogenous hormone levels [75]. The application of salicylic acid can also improve the salt tolerance ability of plants [76]. Here, the protein–protein interaction results showed that *AtBASS2*, the homologous gene of *BrBASS2*, interacted with Nhd1. In *O. sativa*, Nhd1 has been found to alleviate the toxic effects of salt stress on crops by activating nitrogen transporters to regulate root growth and nitrogen uptake [77,78]. When *A*. *thaliana* is subjected to salt stress, the chloroplast Na content of Atnhd1 mutants increases considerably, resulting in the pronounced impairment of photosynthetic performance; moreover, high Na levels may reduce the activity of plastid bile acid/sodium co-transporter family protein 2 (BASS2) [39]. In studies of *T. aestivum*, it was found that a pyruvate transporter called *TaBASS2* is a homolog of *BASS2*. By applying a NaCl treatment to *A*. *thaliana* and *T. aestivum*, it was found that *TaBASS2*-overexpressing plants could alleviate the growth inhibition caused by high salinity and enhance the salt tolerance of both plant species [8]. Likewise, we deduced that the homologous gene *BrBASS2* of *BASS2* has a similar function in *Brassica* plants’ response to salt stress.

## 5. Conclusions

In summary, we identified eight *BrBASS* genes in the *B. rapa* genome. By analyzing their sequence features, *cis*-elements, protein-protein interactions, predicted microRNA targets, abiotic stress tolerance, and published data, we further determined that *BrBASS2* may play a key role in regulating tolerance to salt stress, that *BrBASS4* has the greatest potential for regulating the osmotic stress response, and that *BrBASS7* has substantial potential for regulating cold stress tolerance.

## Figures and Tables

**Table 1:** Physicochemical properties of the proteins encoded by *BrBASS* genes.

Gene ID	Gene Name	Chromosome (Chr)	pI	Molecular Weight (Da)	Amino Acid Length (aa)	Subcellular Location	*A. thaliana* ID	*A. thaliana* Name
*Bra000760*	*BrBASS1*	A03: 12,923,140–12,925,015	9.55	44,669.80	408	chloroplast, nucleus	*AT4G12030*	*AtBASS5*
*Bra005087*	*BrBASS2*	A05: 3,364,680–3,367,077	9.05	43,526.39	409	chloroplast, mitochondrion	*AT2G26900*	*AtBASS2*
*Bra007221*	*BrBASS3*	A09: 28,054,866–28,058,262	9.87	45,302.39	424	chloroplast, plastid, endoplasmic reticulum	*AT3G56160*	*AtBASS4*
*Bra015123*	*BrBASS4*	A07: 3,317,378–3,319,030	9.19	46,419.66	440	chloroplast	*AT3G25410*	*AtBASS3*
*Bra017101*	*BrBASS5*	A04: 16,605,382–16,607,804	8.95	43,853.78	411	chloroplast, plastid, endoplasmic reticulum	*AT2G26900*	*AtBASS2*
*Bra019352*	*BrBASS6*	A03: 24,753,421–24,755,229	9.68	44,509.97	414	chloroplast, plastid, endoplasmic reticulum, mitochondrion	*AT4G22840*	*AtBASS6*
*Bra029434*	*BrBASS7*	A09: 18,128,373–18,130,421	9.12	43,806.66	403	chloroplast, nucleus, cytosol, plastid	*AT4G12030*	*AtBASS5*
*Bra035047*	*BrBASS8*	A07: 21,938,713–21,941,116	9.05	22,989.98	212	chloroplast, mitochondrion, vacuolar	*AT1G78560*	*AtBASS1*

**Table 2:** Details of BrBASSs and targeted miRNAs.

MiRNAs	Targeted BrBASSs Genes	
Bra-miR158-3p	*BrBASS6*	
Bra-miR400-3p	*BrBASS8*	
Bra-miR5720	*BrBASS3*	
Bra-miR5721	*BrBASS3*	
Bra-miR5725	*BrBASS7*	
Bra-miR5726	*BrBASS5*	
Bra-miR9565-3p	*BrBASS7*	
Bra-miR396-3p	*BrBASS4*	*BrBASS7*
Bra-miR162-5p	*BrBASS3*	*BrBASS6*

## Data Availability

All the data that support the findings of this study are available in the paper and its Supplementary Materials published online.

## Supplementary Materials

The following supporting information can be downloaded at https://www.mdpi.com/article/10.3390/cimb46070396/s1.
