# Supplementary figures and images for: Genome-Wide Identification and Expression Analysis of BrBASS Genes in Brassica rapa Reveals Their Potential Roles in Abiotic Stress Tolerance
Source: Curr Issues Mol Biol. 2024 Jun 28;46(7):6646–64. doi: 10.3390/cimb46070396 (PMC11275500; doi:10.3390/cimb46070396)

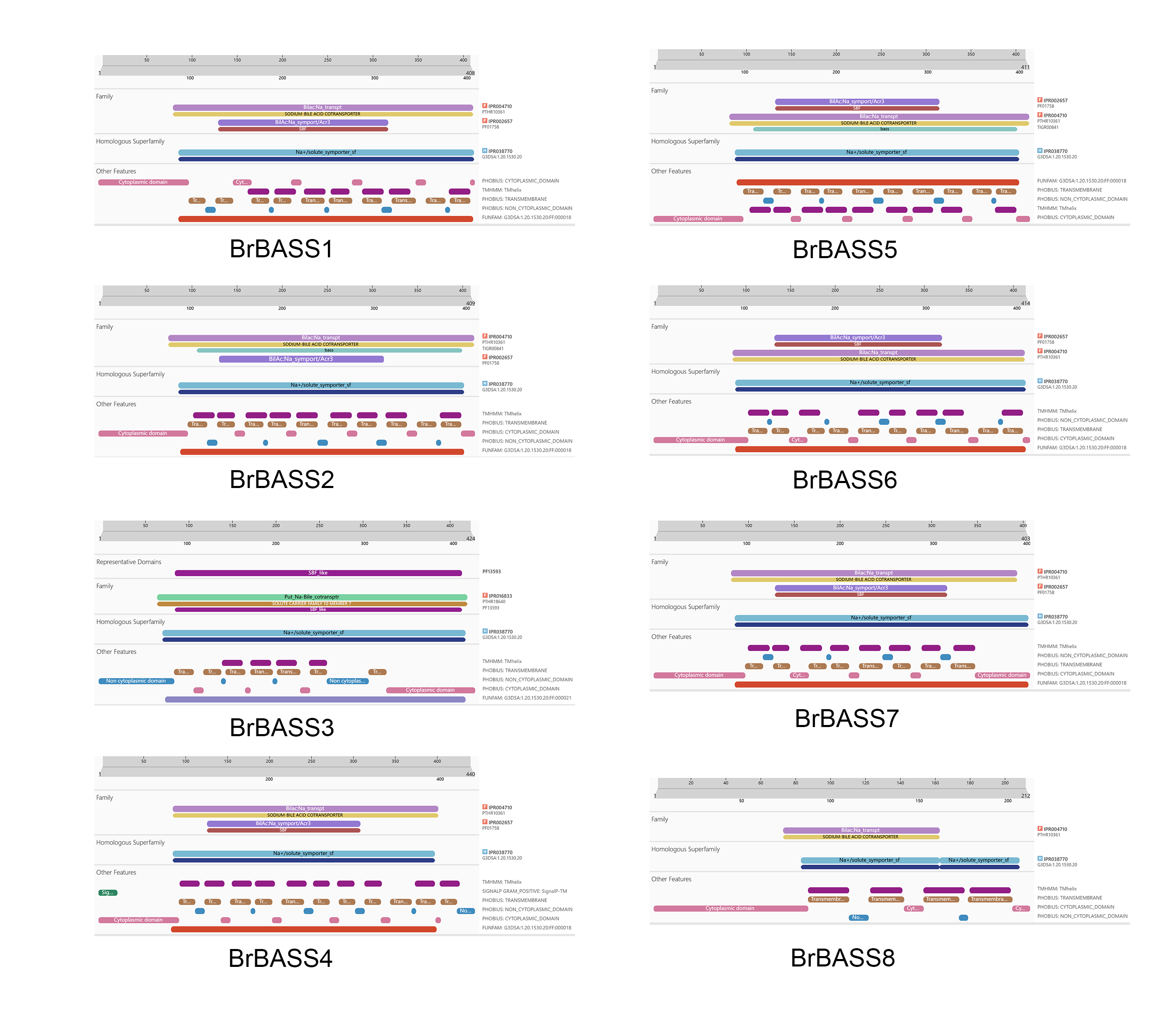

Supplement: Supplementary file 1 [file cimb-46-00396-s001.zip › Figure S1.png]
